# Colonic Diverticulosis and Non-Alcoholic Fatty Liver Disease: Is There a Connection?

**DOI:** 10.3390/medicina58010038

**Published:** 2021-12-27

**Authors:** Ivana Pantic, Sofija Lugonja, Nina Rajovic, Igor Dumic, Tamara Milovanovic

**Affiliations:** 1 Clinic of Gastroenterology and Hepatology, University Clinical Center of Serbia, 11000 Belgrade, Serbia; ilic.ivana04@gmail.com; 2 Department of Internal Medicine, Division of Gastroenterology, General Hospital “Djordje Joanovic”, 23000 Zrenjanin, Serbia; prolesofija@gmail.com; 3 Faculty of Medicine, Institute for Medical Statistics and Informatics, University of Belgrade, 11000 Belgrade, Serbia; nina94rajovic@gmail.com; 4 Mayo Clinic College of Medicine and Science, Rochester, MN 55905, USA; Dumic.Igor@mayo.edu; 5 Division of Hospital Internal Medicine, Mayo Clinic Health System, Eau Claire, WI 54702, USA; 6 Department of Internal Medicine, Faculty of Medicine, University of Belgrade, 11000 Belgrade, Serbia

**Keywords:** non-alcoholic fatty liver disease, colonic diverticulosis, metabolic syndrome

## Abstract

*Background and Objectives:* The development and severity of colonic diverticulosis and non-alcoholic fatty liver disease (NAFLD) has been associated with several components of metabolic syndrome (MetS). Therefore, this study aimed to evaluate a possible connection between NAFLD, colonic diverticulosis, and MetS. *Materials and Methods:* This retrospective study included patients diagnosed with diverticulosis between January 2017 and December 2019. Data regarding the patient demographics, Diverticular Inflammation and Complication Assessment (DICA) score and category, disease localization, hepatic steatosis, blood pressure, comprehensive metabolic panel, need for colonic surgery, and co-morbidities were collected from medical records. *Results:* A total of 407 patients with a median age of 68 years (range, 34–89 years) were included (male: 53.81%). The majority was diagnosed with left-sided diverticulosis (*n* = 367, 90.17%) and an uncomplicated disease course (DICA category 1, *n* = 347, 85.3%). Concomitant hepatic steatosis was detected in 47.42% (*n* = 193) of patients. The systolic blood pressure, triglycerides, total cholesterol, C-reactive protein (CRP), and fasting glucose were higher in the NAFLD group (*p* < 0.001, *p* < 0.001, *p* < 0.001, *p* < 0.001, and *p* < 0.001, respectively). A higher prevalence of hypertension (HTA), type 2 diabetes mellitus (T2DM), and hypothyroidism was noted in the same group of patients (*p* < 0.001, *p* < 0.001, and *p* = 0.008, respectively). High-density lipoprotein cholesterol was lower in patients with more severe forms of diverticulosis (DICA category 2 and 3), while CRP levels were significantly higher (*p* = 0.006 and *p* = 0.015, respectively). HTA and NAFLD were more common in patients with more severe forms of colonic diverticulosis (*p* = 0.016 and *p* = 0.025, respectively). Using a multivariate logistic regression, the DICA score, CRP, total cholesterol, HTA, and hypothyroidism were identified as discriminating factors for the presence of hepatic steatosis. *Conclusion:* Components of metabolic dysregulation were prominent in patients diagnosed with colonic diverticulosis and concomitant hepatic steatosis. HTA, T2DM, and hypothyroidism were more frequently observed in this group. Hepatic steatosis was more commonly detected in more severe forms of colonic diverticulosis.

## 1. Introduction

The prevalence of commonly occurring colonic diverticulosis increases significantly with age, ranging from 10% to 60% [1]. Recent studies have suggested that some components of metabolic syndrome (MetS) such as central obesity, type 2 diabetes mellitus (T2DM), and arterial hypertension (HTA) may contribute to the development and complications related to colonic diverticulosis [2,3,4,5]. Although non-alcoholic fatty liver disease (NAFLD) was previously considered a “hepatic manifestation” of MetS, increasing data implies that it is a systemic disease. NAFLD often occurs together with several other multi-organ disorders including cardiovascular diseases, chronic kidney disease (CDK), T2DM, polycystic ovarian syndrome, psoriasis, malignancies, osteoporosis, and central obesity [6,7,8]. The term metabolic dysfunction-associated fatty liver disease (MAFLD) that has recently been introduced in the literature is defined as the presence of hepatic steatosis (which is confirmed by performing a liver biopsy, imaging methods, or blood biomarkers) and additionally one of these three possible criteria: (1) being overweight or obese, (2) the presence of T2DM, or (3) evidence of metabolic dysregulation [9]. Therefore, the relationship between NAFLD and MetS can be considered reciprocal.

Although left-sided colonic diverticulosis is typically observed in the Western population, in the Asian population it is more commonly located on the right side [1,10]. Emerging evidence suggests an important role of visceral adipose tissue (VAT) in the pathogenesis of colonic diverticulosis and its complications [11,12]. This observation has led many researchers to establish and re-evaluate the potential relationship between colonic diverticulosis and MetS [2,13]. Additionally, chronic low-grade inflammation, a consequence of adipose tissue activity, is thought to play an important role in the pathogenesis of extrahepatic manifestations of NAFLD [7]. The main causes of protracted inflammation in NAFLD are an altered gut microbiota composition and function along with VAT accumulation, which result in an imbalance in pro- and anti-inflammatory cytokines [14]. Previous research has associated VAT accumulation and chronic low-grade inflammation in colonic diverticulosis [4,15].

NAFLD could be considered the cause and the consequence of HTA [8]. Patients having poorly regulated HTA experience a higher risk of developing asymptomatic colonic diverticulosis [3]. Additionally, patients diagnosed with T2DM are estimated to have a higher risk of colonic diverticulosis, diverticular bleeding, and colectomy due to the inability to control the diverticular bleeding. Adequate glucose-regulation is considered a protective factor against diverticular bleeding [5]. Therefore, the complicated relationship between MetS, NAFLD, and colonic diverticulosis should be carefully elucidated.

This study evaluated several components of MetS to determine the possible association between hepatic steatosis and the severity of colonic diverticulosis.

## 2. Materials and Methods

This retrospective cohort study included colonic diverticulosis patients who were diagnosed and treated from January 2017 to December 2019 at the General Hospital “Djordje Joanovic” in Zrenjanin, Serbia. At the time of the colonic diverticulosis diagnosis, the Diverticular Inflammation and Complication Assessment (DICA) score was calculated for each patient [16]. Additionally, a computed tomography of the abdomen and abdominal ultrasonography were performed on all patients. Underage patients as well as pregnant and breastfeeding women were excluded. The data regarding the demographics, DICA score and category, location of the disease, presence of hepatic steatosis, blood pressure at the time of admission, an initial comprehensive metabolic panel, previous colonic surgery, and co-morbidities (HTA, T2DM, and CDK) were collected from the patients’ medical records.

The study was approved by the Ethics Committee of the General Hospital “Djordje Joanovic”. Since the study was purely observational and utilized information from routine clinical practice, the Ethics Committee waived the requirement for informed consent.

### Statistical Analysis

The absolute and relative frequencies of the categorical data were presented. The Kolmogorov–Smirnov test was used to measure the normality of the data distribution for continuous variables. All the non-normally distributed continuous variables were presented as the median and range. Categorical variables were appropriately analyzed by the Chi-square test or Fisher’s exact test. The Mann–Whitney–Wilcoxon or Kruskal–Wallis test by rank were applied for continuous variables without a normal distribution analysis, where appropriate. Univariate and multivariate logistic regression analyses were used to analyze the factors influencing the presence of hepatic steatosis. In all analyses, the significance level was set at 0.05. The statistical analysis was performed using IBM SPSS statistical software (SPSS for Windows, release 25.0, SPSS, Chicago, IL, USA).

## 3. Results

A total of 407 patients were included in the study. The median age of patients was 68 years (range 34–89 years), and 53.8% were male. The majority of patients were diagnosed with left-sided diverticulosis (90.17%), 4.18% of them were diagnosed with right-sided diverticulosis, and 0.49%of them were diagnosed with both-sided diverticulosis. The diverticula extended throughout the entire colon in 5.16% of patients. According to the DICA score, 347 patients (85.3%) were considered DICA category 1, and 14.7% of the patients were considered DICA category 2 and 3. Due to the inability to control complications of the disease, hemicolectomy was performed on six patients (1.47%). Concomitant hepatic steatosis was detected in 47.42% of colonic diverticulosis patients.

The characteristics of the patients with and without hepatic steatosis are presented in Table 1.

In patients with colonic diverticulosis and concomitant hepatic steatosis, the fasting glucose, total cholesterol, triglycerides, CRP levels, systolic blood pressure value, as well as AST, ALT, ALP and GGT levels were all more prominent compared to those without hepatic steatosis. Additionally, HTA, T2DM, and hypothyroidism were observed to be more frequent in patients with hepatic steatosis, which was of statistical significance.

When the same variables were compared for the severity of colonic diverticulosis, it was observed that HDL cholesterol was lower in more severe forms of diverticulosis (DICA category 2 and 3), while CRP and ALP levels were significantly higher (*p* = 0.006, *p* = 0.015, and *p* = 0.029, respectively). Hypertension was more common in patients with more severe forms of colonic diverticulosis (DICA category 2 and 3) (*p* =0.016) (Table 2). Hepatic steatosis was more frequently detected in more severe forms of colonic diverticulosis (*p* =0.025) (Table 3).

The univariate logistic regression showed that potential factors influencing the presence of hepatic steatosis were a higher DICA score, total cholesterol, triglycerides, and CRP, as well as the presence of hypertension, diabetes mellitus type 2, and hypothyroidism. The multivariate logistic regression showed that for one unit enlargement in the DICA score, total cholesterol, and CRP, the chance for the presence of hepatic steatosis enlarged 1.29, 1.40, and 1.01 times, respectively. Additionally, the presence of HTA and hypothyroidism enlarged the chance for the presence of hepatic steatosis 2.18 and 1.93 times, respectively (Table 4).

## 4. Discussion

In agreement with the various studies that addressed the clinical characteristics of colonic diverticulosis, the median age of the patients in this group was 68 years (range 34–89), with a similar distribution between sexes [12,13,17]. Consistent with the results of other European studies [13,18], the majority of the newly diagnosed patients were observed to have an uncomplicated left-sided colonic diverticulosis (90.17%). Interestingly, hepatic steatosis was detected in almost half of these patients (47.42%). Although there is a lack of evidence regarding the interrelation between colonic diverticulosis and NAFLD, it has been suggested that these two disorders commonly co-exist and share several traits of MetS [19]. Kempiński et al. reported concomitant colonic diverticulosis as the second most frequent gastrointestinal disease along with NAFLD (after gastroesophageal reflux disease) that was more prevalent in the patients’ group than in the controls (23.7% vs. 15.8%; *p* < 0.005) [20], while Bae et al. reported a higher overall prevalence of fatty liver in colonic diverticulosis patients (69.7%) as compared to the results of this study [21]. Additionally, the evaluated characteristics of metabolic dysregulation (fasting glucose, systolic blood pressure, triglycerides, total cholesterol, and CRP) were more prominent in colonic diverticulosis patients in whom hepatic steatosis was detected. Furthermore, HTA, T2DM, and hypothyroidism were also more frequently observed in these patients. Although numerous studies have evaluated the effect of MetS components on colonic diverticulosis and NAFLD independently, studies addressing these issues at the same time are still lacking. The prospective study by Teixeira et al. reported a positive association between colonic diverticulosis and an increased waist circumference, elevated blood pressure, as well as hyperlipidemia, whereas no difference was noted regarding the prevalence of T2DM in patients with or without diverticulosis. Additionally, MetS was frequently observed in these patients. The multivariate analysis associated colonic diverticulosis with patients’ age and increased waist circumference, indicating that HTA, T2DM, and dyslipidemia are not independent risk factors for the development of colonic diverticulosis [13]. Another study by Yeo et al. reported that the participants diagnosed with HTA were more likely than those without HTA to be diagnosed with colonic diverticulosis. Several components of metabolic dysregulation (higher triglycerides, uric acid, fasting glucose, and creatinine levels) were more prominent in colonic diverticulosis patients as well [3]. Although another study suggested an inverse relationship between colonic diverticulosis and T2DM [2], it is important to stress that T2DM is an important risk factor for diverticular bleeding [5]. According to the results of this study, when the aforementioned variables were compared with respect to the severity of colonic diverticulosis, differences were observed in HDL cholesterol and CRP median values between DICA groups 1, and groups 2 and 3. Additionally, we observed that HTA was more common in more severe forms of colonic diverticulosis. A recent longitudinal study provided a more thorough insight into the complexity of NAFLD. Ma et al. reported that subjects diagnosed with NAFLD were more likely to have MetS, lower HDL, higher triglycerides, and increased fasting glucose levels. The results of the study showed that participants with a fatty liver had greater odds of incident HTA and T2DM [8]. Interestingly, in this study, hepatic steatosis was observed to be more prevalent in more severe forms of colonic diverticulosis, which was previously unreported to the best of the authors’ knowledge. Additionally, in this study, using a multivariate logistic regression, the DICA score, CRP, total cholesterol, and presence of HTA and hypothyroidism were identified as key discriminating factors for the presence of hepatic steatosis in patients with colonic diverticulosis. Bae et al. reported a significant association of the waist-to-hip ratio as well as a moderate and severe fatty liver with the risk of asymptomatic diverticulosis. Although the study designs differ to a great extent, the possible role of central obesity and NAFLD in the pathogenesis of asymptomatic forms of the disease is in agreement with the results of this study [21].

### Limitations of the Study

This is one of the first single-center, retrospective, and cross-sectional studies to address a possible relationship between colonic diverticulosis and NAFLD, while stressing an important role of MetS in both of these disorders. The limitations of the study are associated with its retrospective nature. Several crucial factors including anthropometric data and risk factors (smoking status, alcohol consumption, and dietary fiber intake) could not be incorporated into the analyses due to the study design. We are also aware that NAFLD was estimated based on imaging and that no liver biopsy was performed. Furthermore, Cohen’s kappa coefficient cannot be employed. The exams were done by a single experienced gastroenterologist and radiologist.

## 5. Conclusions

This study showed that components of metabolic dysregulation are more prominent in patients diagnosed with colonic diverticulosis and concomitant hepatic steatosis. HTA, T2DM, and hypothyroidism were more frequently observed in the same group of patients. Fatty liver was commonly observed in more severe forms of colonic diverticulosis. The DICA score, CRP, total cholesterol, and presence of HTA and hypothyroidism were identified as key discriminating factors for the presence of hepatic steatosis in patients with colonic diverticulosis.

## Figures and Tables

**Table 1:** Characteristics of the study population concerning the concomitant presence of hepatic steatosis.

Variable	Hepatic Steatosis			*p*-Value
	Yes (*n* = 193)	No (*n* = 214)	Total (*n* = 407)	
Age, years (median, range)	69 (36–89)	67.5 (34–86)	68 (34–89)	0.166
Sex, male (*n,* %)	101 (52.33)	118 (55.14)	219 (53.81)	0.570
Fasting glucose, mmol/L (median, range)	6.1 (2.4–15.9)	5.45 (2.8–16.7)	5.9 (2.4–16.7)	<0.001
Systolic blood pressure, mmHg (median, range)	140 (80–240)	140 (85–230)	140 (80–240)	0.036
Diastolic blood pressure, mmHg (median, range)	90 (60–145)	90 (45–145)	90 (45–145)	0.293
Tryglicerides, mmol/L (median, range)	1.96 (0.5–6.67)	1.7 (0.2–13)	1.8 (0.2–13)	<0.001
HDL ^1^ cholesterol, mmol/L (median, range)	1 (0.3–2.46)	1.02 (0.2–2.3)	1 (0.2–2.46)	0.093
Total cholesterol, mmol/L (median, range)	5.7 (1.18–9.8)	4.8 (1.45–10)	5.03 (1.18–10)	<0.001
CRP ^2^, mg/L (median, range)	12 (0.1–418.5)	5 (0.1–211)	8 (0.1–418.5)	<0.001
Hypertension (yes/no, *n* (%))	149/44 (77.2/22.8)	114/100 (53.27/46.73)	263/144 (64.62/35.38)	<0.001
Diabetes mellitus type 2 (yes/no, *n* (%))	126/67 (65.28/34.72)	95/119 (44.39/55.61)	221/186 (54.3/45.7)	<0.001
Hypothyroidism (yes/no, *n* (%))	41/152 (21.24/78.76)	24/190 (11.21/88.79)	65/342 (15.97/84.03)	0.008
AST ^3^, U/L (median, range)	18 (8–187)	15 (6–676)	16 (6–676)	<0.001
ALT ^4^, U/L (median, range)	18 (6–364)	15 (7–259)	17 (6–364)	0.001
ALP ^5^, U/L (median, range)	35 (9–401)	18 (9–703)	24 (9–703)	<0.001
GGT ^6^, U/L (median, range)	26 (5–735)	20 (8–1115)	23 (5–1115)	0.011
Chronic kidney disease, unspecified (yes/no, *n* (%))	9/184 (4.66/95.34)	4/210 (1.87/98.13)	13/394 (3.19/96.81)	0.187

^1^ High-density lipoprotein,^2^ C-reactive protein, ^3^ Aspartate amino-transferase, ^4^ Alanin amino-transferase, ^5^ Alkaline phosphatase, ^6^ Gamma glutamyl transferase.

**Table 2:** Characteristics of the study population concerning the severity of colonic diverticulosis.

Characteristic	DICA Category ^1^		*p*
	1 (*n* = 347)	2 + 3 (*n* = 60)	
Age, years (median, range)	68 (34–89)	70 (45–86)	0.343
Sex (male/female, *n* (%))	185/162 (53.31/46.69)	34/26 (56.7/43.3)	0.631
Fasting glucose, mmol/L (median, range)	5.8 (2.4–16.7)	6.09 (2.8–12.3)	0.596
Systolic blood pressure, mmHg (median, range)	140 (90–240)	130 (80–190)	0.335
Diastolic blood pressure, mmHg (median, range)	90 (60–145)	80 (45–100)	0.196
Tryglicerides, mmol/L (median, range)	1.8 (0.2–13)	1.53 (0.50–5.26)	0.244
HDL ^2^ cholesterol, mmol/L (median, range)	1.01 (0.2–2.46)	0.92 (0.30–1.86)	0.006
Total cholesterol, mmol/L (median, range)	5.03 (1.18–10)	5.01 (1.45–9.20)	0.769
CRP ^3^, mg/L (median, range)	7 (0.1–418.5)	14.95 (0.4–306.7)	0.015
Hypertension (yes/no, *n* (%))	216/131 (62.3/37.7)	47/13 (78.3/21.7)	0.016
Diabetes mellitus type 2 (yes/no, *n* (%))	185/162 (53.3/46.7)	36/24 (60.0/40.0)	0.337
Hypothyroidism (yes/no, *n* (%))	56/291 (16.1/83.9)	9/51 (15.0/85.0)	0.824
AST ^4^, U/L (median, range)	16 (6–676)	16.5 (9–170)	0.343
ALT ^5^, U/L (median, range)	16 (6–364)	18 (7–220)	0.266
ALP ^6^, U/L (median, range)	22 (9–703)	33.5 (9–357)	0.029
GGT ^7^, U/L (median, range)	23 (5–1115)	26.5 (7–735)	0.318
Chronic kidney disease, unspecified (yes/no, *n* (%))	9/338 (2.59/97.41)	4/56 (6.7–93.3)	0.098

^1^ Diverticular Inflammation and Complication Assessment, ^2^ High-density lipoprotein, ^3^ C-reactive protein, ^4^ Aspartate amino-transferase,^5^ Alanin amino-transferase,^6^ Alkaline phosphatase, ^7^ Gamma glutamyl transferase.

**Table 3:** Hepatic steatosis and colonic diverticulosis disease severity.

Variable	Hepatic Steatosis		*p*-Value
	Yes (*n* = 193)	No (*n* = 214)	
DICA ^1^ category			
1 (*n*, %)	155 (80.31)	192 (89.72)	0.025
2 (*n*, %)	33 (17.1)	20 (9.35)	
3 (*n*, %)	5 (2.59)	2 (0.93)	
DICA score (median, range)	3 (1–12)	3 (1–11)	0.004
Hemicolectomia			
Yes (*n*, %)	3 (1.55)	3 (1.4)	1
No (*n*, %)	190 (98.45)	211 (98.6)	

^1^ Diverticular Inflammation and Complication Assessment.

**Table 4:** Predictors of hepatic steatosis.

Predictor	Univariate Logistic Regression				Multivariate Logistic Regression			
	B	OR	95%CI OR	*p*	B	OR	95%CI OR	*p*
DICA ^1^, points	0.249	1.282	1.09–1.51	0.003	0.260	1.296	1.09–1.54	0.004
Hypertension, yes	1.089	2.970	1.93–4.57	<0.001	0.779	2.179	1.35–3.51	0.001
T2DM ^2^, yes	0.857	1.356	1.58–3.52	<0.001	0.400	1.492	0.95–2.35	0.083
Hypothyroidism, yes	0.759	2.135	1.24–3.69	0.007	0.660	1.935	1.07–3.49	0.028
Total cholesterol	0.369	1.447	1.27–1.65	<0.001	0.340	1.404	1.20–1.64	<0.001
Triglycerides	0.318	1.374	1.14–1.65	0.001	0.092	1.096	0.89–1.35	0.381
C-reactive protein	0.011	1.011	1.01–1.02	0.001	0.010	1.010	1.00–1.02	0.004

^1^ Diverticular Inflammation and Complication Assessment, ^2^ Type 2 diabetes mellitus.

## Data Availability

All data is available from the corresponding author upon request.
